# Glycosaminoglycan Analysis of FFPE Tissues from Prostate Cancer and Benign Prostate Hyperplasia Patients Reveals Altered Regulatory Functions and Independent Markers for Survival

**DOI:** 10.3390/cancers14194867

**Published:** 2022-10-05

**Authors:** Gábor Tóth, Simon Sugár, Domonkos Pál, Kata Dorina Fügedi, László Drahos, Gitta Schlosser, Csilla Oláh, Henning Reis, Ilona Kovalszky, Tibor Szarvas, Lilla Turiák

**Affiliations:** 1 MS Proteomics Research Group, Research Centre for Natural Sciences, Magyar Tudósok Körútja 2, H-1117 Budapest, Hungary; 2 Doctoral School of Pharmaceutical Sciences, Semmelweis University, Üllői Út 26, H-1085 Budapest, Hungary; 3 Faculty of Chemical Technology and Biotechnology, Budapest University of Technology and Economics, Szt. Gellért Tér 4, H-1111 Budapest, Hungary; 4 MTA-ELTE Lendület Ion Mobility Mass Spectrometry Research Group, Eötvös Loránd University, Pázmány Péter Sétány 1/A, H-1117 Budapest, Hungary; 5 Department of Urology, University Medicine Essen, University of Duisburg-Essen, Hufelandstraße 55, 45147 Essen, Germany; 6 Institute of Pathology, University Medicine Essen, University of Duisburg-Essen, Hufelandstraße 55, 45147 Essen, Germany; 7 Dr. Senckenberg Institute of Pathology (SIP), University Hospital Frankfurt, Goethe University Frankfurt, Theodor-Stern-Kai 7, 60590 Frankfurt am Main, Germany; 8 Department of Pathology and Experimental Cancer Research, Semmelweis University, Üllői Út 26, H-1085 Budapest, Hungary; 9 Department of Urology, Semmelweis University, Üllői Út 26, H-1085 Budapest, Hungary

**Keywords:** glycosaminoglycan, heparan sulfate, chondroitin sulfate, prostate cancer, benign prostate hyperplasia, HPLC, mass spectrometry, proteoglycan, glycomics

## Abstract

**Simple Summary:**

Prostate cancer is one of the most frequent cancer types among men. A better understanding of the involved molecular mechanisms is still necessary for the improvement of therapeutic decision making. Glycosaminoglycan disaccharides were investigated on biopsies taken from patients with prostate cancer and benign prostate hyperplasia. We found that the quantity and sulfation of chondroitin sulfate chains significantly differed between benign prostate hyperplasia and the distinct risk groups of prostate cancer. Doubly and triply sulfated heparan sulfate disaccharides were identified as the independent biomarkers for survival.

**Abstract:**

Prostate cancer is one of the most frequent cancer types among men. Several biomarkers and risk assessment methods are already available; however, enhancing their selectivity and sensitivity is still necessary. For improving therapeutic decisions, both basic and clinical research studies are still ongoing for a better understanding of the underlying molecular mechanisms. The enzymatic digests of heparan sulfate (HS) and chondroitin sulfate (CS) chains were investigated in tissue samples taken from patients with prostate cancer (PCa) and benign prostate hyperplasia (BPH) with the HPLC–MS methodology. None of the HS species analyzed showed correlating alterations with currently used markers such as clinical stage, Gleason score, or prostate-specific antigen (PSA) level. The total quantity and sulfation motifs of CS were both significantly different among BPH and different risk groups of PCa. Furthermore, the cancer-specific survival of patients can be predicted based on the levels of non-sulfated and doubly sulfated CS disaccharides as well as the total HS content and the doubly and triply sulfated HS disaccharide ratios. These disaccharide ratios proved to be independent markers from clinical parameters. Further investigations of glycosaminoglycan motifs were proposed for the validation of the results on independent patient cohorts as well.

## 1. Introduction

Prostate cancer (PCa) is among the most prevalent cancer types among men worldwide [1]. In recent decades, its mortality rate decreased, due to prostate-specific antigen (PSA) screening, advances in therapeutic strategies and drugs, as well as the introduction of several other markers guiding therapeutic decisions [2]. Current biomarkers for incidence and progression estimation include proenzyme PSA (proPSA), the ratio of free and bound PSA, the four-Kallikrein panel, genetic alterations (e.g., *BRCA1/2*), as well as mRNAs (e.g., for *AZGP1, TPM2, TPX2, PCA3,* and *ERG* genes) from tissues and urinary extracellular vesicles [3]. However, the decrease in mortality rate goes hand in hand with overdiagnosis, leading to unnecessarily high numbers of radical surgeries. A significant part of these interventions could be substituted with active surveillance, thus avoiding unpleasant side effects. Therapeutic decisions are made based on several clinical and pathological factors, such as before-treatment PSA level, the clinical T-stage, the Gleason score, and the therefrom-derived International Society of Urological Pathology (ISUP) grade groups (WHO/ISUP grade groups). From the above-mentioned factors, a variety of PCa risk stratification methods (e.g., Cancer of the Prostate Risk Assessment (CAPRA) and D’Amico scores) have been reported, with various endpoints, such as relapse-free survival, overall survival, and cancer-specific survival [4]. The prognostic value of these methods is limited and ignores the biological heterogeneity of PCa. Therefore, a better understanding of the molecular alterations accompanying prostate malfunctions is necessary, as well as a search for novel biomarkers. Ideal biomarkers should have high sensitivity and specificity to identify PCa, including in high-risk cases. PSA screening is very sensitive; however, it lacks specificity when differentiating between benign prostate hyperplasia (BPH) and PCa [5].

The molecular family of proteoglycans (PGs) can serve as potential new clinical markers. PGs are biomolecules containing one or more glycosaminoglycan (GAG) chains covalently attached to defined core proteins [6]. They can mainly be found in the extracellular matrix, on the cell surface, and in the granules found in the cytoplasm, thus exerting essential signaling functions [6]. The type, amount, and structure of the GAG chains play an important role in defining the physicochemical properties and biological functions of PGs along with the type of the core proteins. Therefore, their organization between distinct biological conditions is worth investigating. The two most prevalent classes of GAGs in tissues are chondroitin sulfate/dermatan sulfate (CS/DS) and heparan sulfate (HS). CS consists of the alternating units of *N*-acetylgalactosamine (GalNAc) and glucuronic acid (GlcA), while HS chains are built up of *N*-acetylglucosamine (GlcNAc) and glucuronic/iduronic acid (IdoA). The most common sulfation positions on CS chains are 4-*O* and/or 6-*O* positions of the GalNAc residues [3]. HS chains are subjected to a more complex sulfation cascade. First, *N*-deacetylase/*N*-sulfotransferase (NDST) enzymes act on the given domains of GlcNAc residues to generate *N*-sulfation, and then these domains are acted on by an epimerase to form IdoA residues. The subsequent sulfation reactions include the 2-*O*-sulfation of both IdoA and GlcA residues, the next 6-*O*-sulfation, and (rarely) the 3-*O*-sulfation of GlcNAc and GlcNS residues. GAG chains are responsible for cellular signaling and recognition, governed by the size and the sulfation pattern of the respective chains [7,8,9,10]. Alterations in the ratio of the differentially sulfated disaccharide building blocks may be descriptive of various diseases, e.g., sulfation pattern changes have been observed between healthy and cancerous tissues [11,12,13]. This structural variability also appears on the level of proteoglycans influencing signal transduction. For example, decorin (CSPG) is known to have major tumor suppressor effects, while perlecan (HSPG) plays a central role in both physiological and pathological angiogenesis [14]. Several functions of PGs have already been observed specifically in PCa as well; however, most of the structural and functional changes on both the PG and GAG levels remain to be investigated [15].

In the present study, we performed high-performance liquid chromatography–mass spectrometry (HPLC–MS)-based GAG disaccharide analysis for the investigation of BPH and PCa tissues. We analyzed the CS and HS content and the structure of the tissue in correlation with the different types of operation and risk assessment groups. Finally, Kaplan–Meier and multivariate analyses were performed to identify potential new markers for the assessment of survival probability.

## 2. Materials and Methods

### 2.1. Chemicals and Reagents

LC–MS grade solvents were purchased from VWR Hungary (Debrecen, Hungary). Ammonium formate, ammonium acetate, formic acid, Tris-HCl, Ca(OH)_2_, glycerol, and chondroitinase ABC were from Sigma (Budapest, Hungary). Heparan sulfate and chondroitin sulfate disaccharide standards and heparin lyase I-II-III enzymes were purchased from Iduron Ltd. (Cheshire, UK).

### 2.2. Patients Selection

Formalin-fixed and paraffin-embedded (FFPE) tissue samples were retrospectively collected from 77 patients who underwent prostate surgery at the Department of Urology, the University of Duisburg–Essen, between 1991 and 2004. The full study cohort included 16 BPH samples and 61 PCa samples (8 patients with palliative transurethral resection (pTURP) surgery and 53 with radical prostatectomy (RPE)). PCa samples were stratified into risk groups according to the Gleason grading system (version 2014). Gleason grade group 1 defined low-risk PCa, grade groups 2 and 3 were considered the intermediate risk group, while grade groups 4 and 5 were regarded as high risk. The CS study contained 14 BPH, 21 low-risk PCa, 19 intermediate-risk PCa, and 14 high-risk PCa samples. The HS study contained 14 BPH, 20 low-risk PCa, 17 intermediate-risk PCa, and 17 high-risk PCa samples. Patients’ characteristics are given in detail in Table S1. The differences in the number of patients in the CS and HS cohorts are caused by the fact that the samples with sample preparation or injection errors were discarded from the respective cohort. We applied cancer-specific survival (CSS) as the study endpoint, which was calculated as the time between surgery and PCa-related death; death from other causes and incomplete observations (last update 10/2020) were treated as censored data. Considering the relatively slow disease progression of clinically localized PCa treated by RPE, a very long follow-up period is needed to evaluate the prognostic value of biomarkers. In our study, the median follow-up time was 186 months with a mean of 185.5 months and a maximum of 336.2 months. As tumor-specific death in a clinically localized PCa cohort occurs in a relatively low rate of RPE-treated patients, it limited our possibilities of patient selection for multivariate CSS analyses. Ethical approval was issued by the University of Duisburg–Essen Ethics Committee under registration number 21-9991-BO. 

### 2.3. FFPE Tissue Preparation

**Figure 1:**
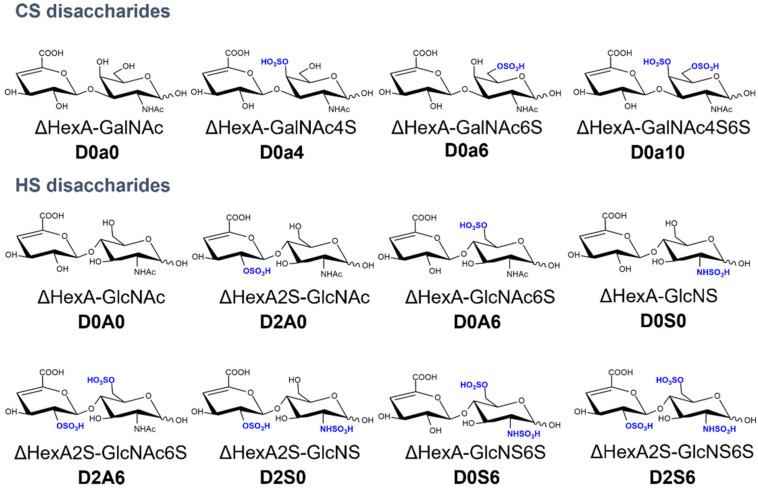
The chemical structures, traditional names, and Lawrence codes of the analyzed disaccharides.

Deparaffination was followed by antigen retrieval in 100 mM Tris-HCl (pH = 7.6) for 30 min. The tumorous regions of the tissues and given parts of the BPH tissues were selected based on histological staining. The selected parts of unstained tissues were carved with a Gillette blade and digested with on-tissue digestion as described before [12,16]. For heparan sulfate (HS) digestion, a mixture of 5 mU heparin lyase I and 1-1 mU of heparin lyase II and III in 20 mM Tris-HCl (pH = 7.6), 2.5 mM Ca(OH)_2_, 10% glycerol solution was added in five hourly portions, and the digestion was run in a humidified box for 48 h. For chondroitin sulfate (CS) digestion, 25 mU of chondroitinase ABC in 20 mM Tris-HCl (pH = 7.6), 2.5 mM NH_4_OAc, 10% glycerol solution was added in five hourly portions, and the digestion was run in a humidified box for 48 h. The resulting HS and CS disaccharides were extracted from the surface via the repeated pipetting of 0.3% ammonia solution. The samples were dried down and purified in the TopTip graphite + C_18_ spin tip SPE system (Glygen corp., Columbia, MD, USA). The investigated disaccharides with their traditional names and Lawrence codes are represented in Figure 1. 

### 2.4. High-Performance Liquid Chromatography–Mass Spectrometry

**Figure A1:**
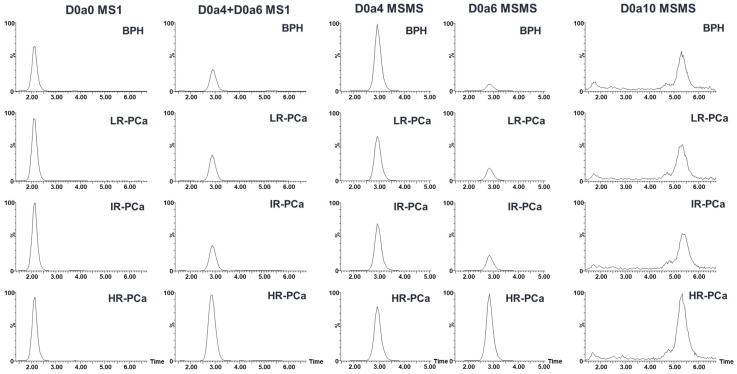
Representative extracted ion chromatograms of CS disaccharide measurements. See Figure 1 for resolving the GAG structure codes.

**Figure A2:**
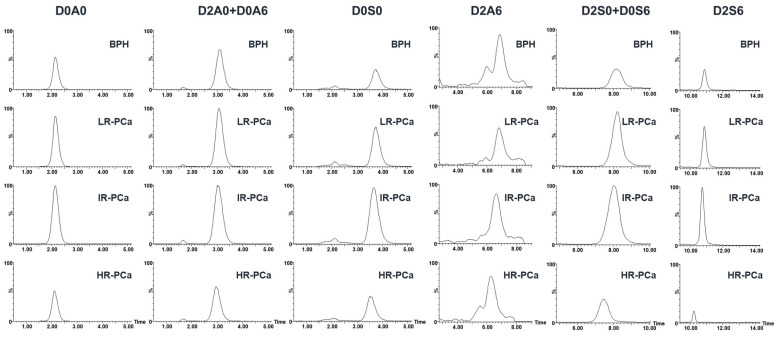
Representative extracted ion chromatograms of HS disaccharide measurements. See Figure 1 for resolving the GAG structure codes.

The HPLC–MS measurements were performed on a Waters Acquity I-class UPLC instrument (Milford, MA, USA) coupled to a Waters Select Series Cyclic Ion Mobility (Milford, MA, USA) mass spectrometer. For the chromatographic separation of CS and HS disaccharides, a self-packed GlycanPac AXH-1 capillary column (250 µm i.d.) was used with the ammonium formate salt gradient methods published before [17,18]. In the low-flow ESI ion source, the capillary voltage was set to 1.9 kV, while the cone voltage was 20 eV, and the temperature was 120 °C. The HS disaccharides were measured in MS1 mode, with the trap collision energy being 6 eV, and the transfer being 3eV. The CS was measured in MS1 and MS/MS modes, where the monosulfated isomer pairs were fragmented with 32 eV in the transfer to determine sulfation positions. Finally, the extracted ion chromatograms were integrated with the TargetLynx add-in of MassLynx software v4.2, Waters Corporation (Milford, MA, USA). The detailed integration method parameters are summarized in Table S2. Chromatogram examples are shown in Appendix A: Figure A1 (representative extracted ion chromatograms of CS disaccharides) and Figure A2 (representative extracted ion chromatograms of HS disaccharides).

### 2.5. Statistical Analysis, Data Visualization

The experimental data were first total area normalized to the sum of the intensities of all measured GAG chains (separately for CS and HS disaccharides). The total abundance of CS and HS was calculated by summing up the signals in one sample without any further normalization step. The normality of each sample group compared (combinations of GAG chains and risk groups) was tested using Shapiro–Wilk tests. Multiple sample comparisons were performed using Kruskal–Wallis tests. In the case of Kruskal–Wallis significant GAG chains, two-sample comparisons were performed. For normally distributed data, equal variance assumptions were checked using F tests. Based on the result, *t*-tests or Welch *t*-tests were used. For non-normally distributed data, Mann–Whitney tests were used. The statistical tests used are summarized in Table S3. All the statistical tests and the survival analysis (survival package) were performed using R 4.0.5 [19] in RStudio 1.4.1106 [20], and boxplots were made with ggplot. Kaplan–Meier plots were made using a web app https://kmplot.com/analysis/ (accessed on 22 July 2022) [21]. For the survival analysis, Cox regression was used (coxph function). Multivariate regression was performed in a pairwise manner for GAG motifs and clinical parameters. FDR control was performed on *p*-values using the Benjamini–Hochberg method. Data visualization was performed in R and Microsoft PowerPoint, and the graphical abstract was created with Biorender.com.

## 3. Results

**Figure 2:**
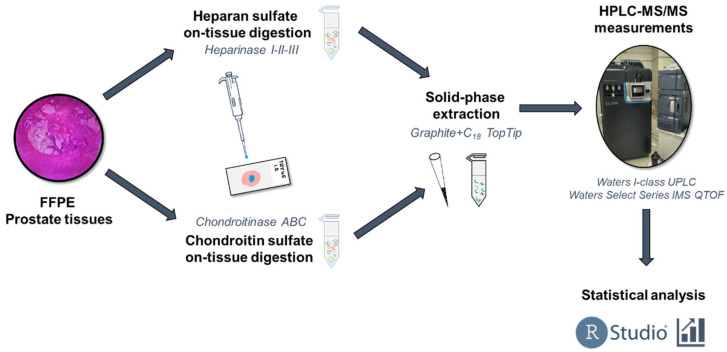
The workflow used in the study.

The chondroitin sulfate and heparan sulfate content and the sulfation pattern of prostate tissues were determined by disaccharide analysis; the workflow can be seen in Figure 2. Then, these levels were analyzed in terms of their influence on CSS, by generating Kaplan–Meier plots and performing log-rank tests. The intensity and sulfation pattern data are discussed based on the risk groups deducted from the Gleason grades. The median and range data based on all the classifications are summarized in Table S4.

### 3.1. Total Abundance of Chondroitin Sulfate and Heparan Sulfate

**Figure 3:**
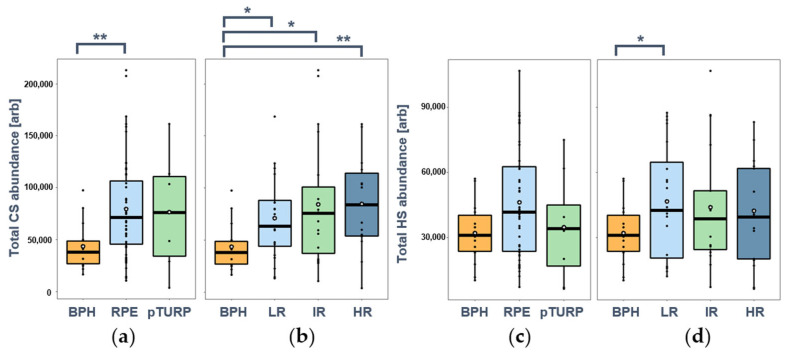
Total abundances of CS and HS chains in BPH and prostate cancer (PCa) tissues: (**a**) CS abundance in BPH and PCa tissues obtained via radical prostatectomy (RPE, *n* = 48) and palliative transurethral resection (pTURP, *n* = 6); (**b**) CS abundance in BPH and PCa tissues from low-risk (LR, *n* = 21), intermediate-risk (IR, *n* = 19), and high-risk (HR, *n* = 14) patients based on Gleason risk stratification; (**c**) HS abundance in BPH and PCa tissues obtained via RPE (*n* = 46) and pTURP (*n* = 8); (**d**) HS abundance in BPH and PCa tissues from LR (*n* = 20), IR (*n* = 17), and HR (*n* = 17) patients based on Gleason risk stratification. (*: *p* < 0.05, **: *p* < 0.01).

First, in analyzing the total abundance of CS (Figure 3a,b), a significant increase was observed in all the cancerous samples compared with the BPH samples. 

When comparing the PCa tissue samples (obtained via RPE or pTURP) with the BPH samples, we observed on average 1.6 times higher CS abundance in both groups. However, the values of the pTURP group showed such high variability that these differences were observed to be statistically significant only in the BPH–RPE comparison (Figure 3a). We then compared the CS abundance between the BPH group and the different risk groups of PCA. We found a 1.61-fold (*p* = 0.0197) increase in the low-risk (LR) PCa, a 1.88-fold (*p* = 0.0361) increase in the intermediate-risk (IR) PCa, and a 1.94-fold (*p* = 0.0032) increase in the high-risk (HR) PCa group. No statistically significant differences were detected between any of the PCa sample groups, although a slightly increasing trend of the median and average values could be observed (Figure 3b).

Comparing the heparan sulfate levels between BPH and RPE samples, a slight increase was observed, but the most progressed pTURP sample group gave almost identical median and mean values to the BPH group (Figure 3c). When looking at the PCa samples according to the risk stratifications, an increase in the mean HS total intensity was observed in cancerous tissues compared with BPH by a factor of 1.32–1.41 (Figure 3d). Although a slightly decreasing trend could be observed with cancer progression, no significant difference was observed in the PCa risk group comparisons regarding the total abundance of HS chains.

### 3.2. Sulfation Motifs of Chondroitin Sulfate

We observed considerable changes in the sulfation pattern between different groups as well. To address this, first, the intensities of individual disaccharides, then the average sulfation, and finally, the ratio of 6-*O*-sulfation to 4-*O*-sulfation (6S/4S ratio hereinafter) are discussed. 

**Figure 4:**
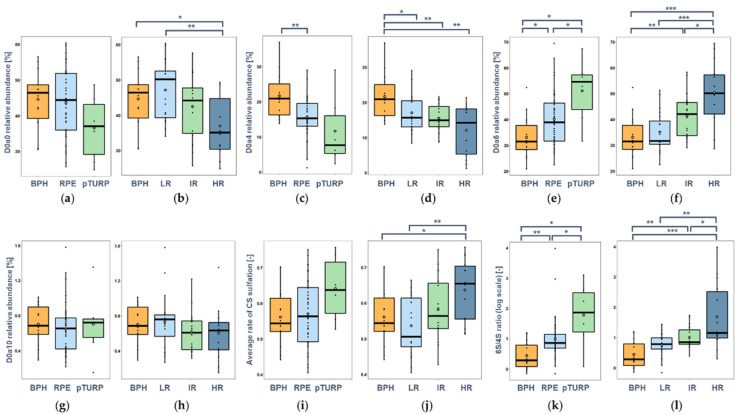
Relative abundance and sulfation characteristics of measured CS disaccharides as a function of the operation type and the risk stratification based on Gleason scores, respectively: (**a**,**b**) D0a0; (**c**,**d**) D0a4; (**e**,**f**) D0a6; (**g**,**h**) D0a10; (**i**,**j**) average rate of CS sulfation; (**k**,**l**) 6S/4S ratio. (*: *p* < 0.05, **: *p* < 0.01, ***: *p* < 0.001). See Figure 1 for resolving the GAG structure codes.

The non-sulfated CS disaccharide (D0a0) was the predominant building block in most of the samples (except for pTURP and high-risk PCa groups). When comparing the different surgery types, we observed that the median relative intensity was ca. 3% less in RPE and ca. 12% less in the pTURP group compared with BPH (Figure 4a). Looking at the PCa risk groups, the relative intensity of D0a0 was higher in LR PCa than in BPH, but a substantial decrease was observed with cancer progression, being 16% lower in the HR than in the LR group (Figure 4b). Significant differences were observed between BPH and HR PCa (*p* = 0.0328) and LR and HR PCa (*p* = 0.0021) groups. 

The largest differences between the sample groups could be observed in the relative intensities of the monosulfated components (Figure 4c–f). The D0a4 disaccharide showed a significantly higher relative intensity in the BPH group than in any of the PCa groups. Comparing the BPH group with the PCa groups with different surgical treatments, a significantly lower level was observed in the RPE group (*p* = 0.0054); however, the pTURP had even lower mean and median values than RPE; thus, the non-significance may be attributed to the low number of pTURP samples investigated and the high intragroup variance observed. Comparing the relative abundance of D0a4 in the case of the different risk groupings, the BPH group showed significantly higher D0a4 levels than the PCa groups (*p* = 0.0151, *p* = 0.0030, *p* = 0.0032, with LR, IR, HR PCa, respectively). 

The greatest number of group pairs with significant differences were observed in the case of the D0a6 disaccharide. Its relative intensity in the BPH group was lower than in any of the PCa groups. In the RPE group, a 1.2-fold (*p* = 0.0153) increase, while in the pTURP surgery group, a 1.7-fold increase (*p* = 0.0159) was observed compared with BPH. Between the two types of surgery, a 1.4-fold significant difference (*p* = 0.0417) was observed in the relative abundance of D0a6 (Figure 4e). Looking at the risk assessment groups, an increasing trend was observed with cancer progression (Figure 4f). HR PCa could be distinguished from BPH and LR PCa well (*p* = 0.00014, and *p* = 0.00029, respectively), with a fold-change around 1.6 in both cases. A significant difference from the intermediate-risk PCa (*p* = 0.0157) was also observed. The difference between BPH and IR PCa was also significant (*p* = 0.0062), but with LR PCa, it was not. The least abundant building block was the doubly sulfated D0a10 disaccharide (Figure 4g,h), and although a similar trend to D0a0 was detected, the differences did not prove to be statistically significant.

Considering these changes in the relative abundance of individual disaccharides, it is worth looking at the sulfation characteristics of CS. The average rate of sulfation was slightly higher on average in the RPE operation group than that in the BPH group. However, a larger interpatient variability was also observed. In the pTURP group, there were on average 14–16% more sulfate groups per disaccharide building block than in the other two groups (Figure 4i). The average rate of sulfation was lower in the low-risk PCa than in the BPH group, but it significantly increased with the increasing risk assessment, being especially high in high-risk PCa with a fold-change of 1.3 (*p* = 0.0022) between the LR and HR groups (Figure 4j). 

As there was an increasing trend in *6-O*-sulfation and a decreasing trend in *4-O*-sulfation, we took a closer look at the ratio of the two disaccharides as well. We could conclude that the 6S/4S ratio showed a large increase in PCa compared with BPH; it was 1.8- and 5.2-fold in RPE and pTURP, respectively (Figure 4k), and 1.7-, 1.8-, and 2.4-fold in LR, IR, and HR PCa, respectively (Figure 4l). These differences were statistically significant in all the cases except between LR and IR PCa. 

### 3.3. Sulfation Motifs of Heparan Sulfate

**Figure 5:**
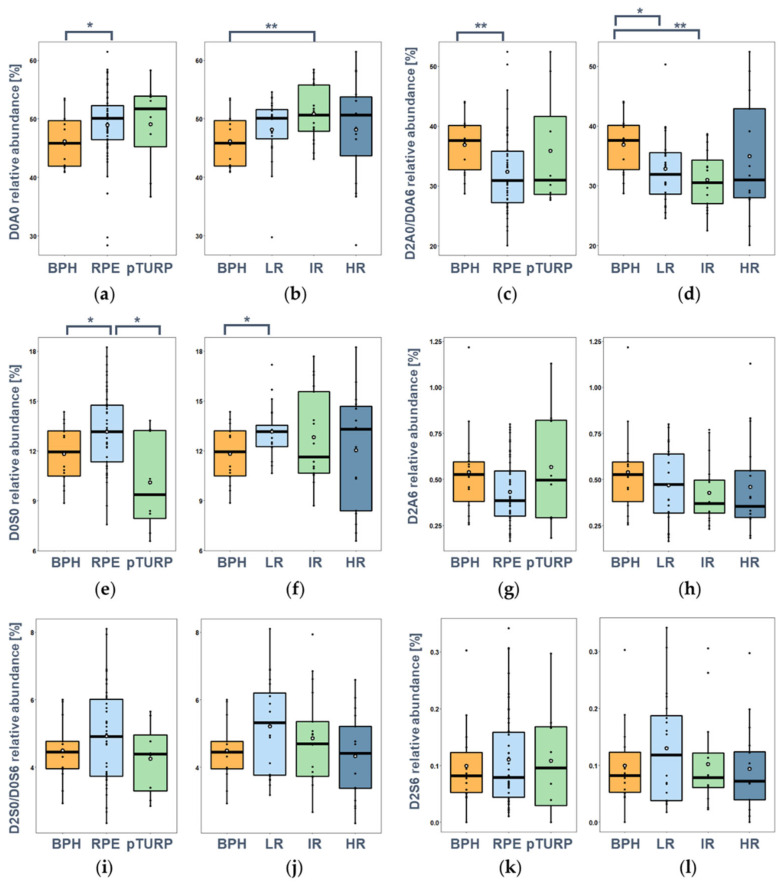
Relative abundance of measured HS disaccharides as a function of the surgery type and the risk stratification based on Gleason scores, respectively: (**a**,**b**) D0A0; (**c**,**d**) D2A0/D0A6; (**e**,**f**) D0S0; (**g**,**h**) D2A6; (**i**,**j**) D2S0/D0S6; (**k**,**l**) D2S6. (*: *p* < 0.05, **: *p* < 0.01). See Figure 1 for resolving the GAG structure codes.

Regarding the sulfation pattern (the relative intensity of HS disaccharides), much smaller differences could be observed than that for CS. The most predominant building block was the non-sulfated D0A0 disaccharide, the mono-O-sulfated isomer pair (D2A0 + D0A6) was present in comparable amounts to D0A0, and the D0S0 mono-*N*-sulfated component showed 4-5-fold lower intensities. The doubly and triply sulfated building blocks were present only in minor amounts; D2A6 and D2S6 were usually under 1% and the D2S0 + D0S6 isomer pair around 1–5% (Figure 5). 

**Figure A3:**
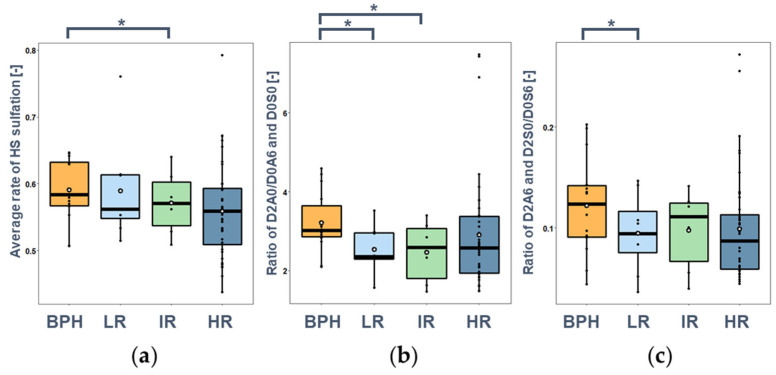
Sulfation characteristics of HS chains as a function of the risk stratification based on Gleason scores: (**a**) average rate of HS sulfation; (**b**) O/N ratio of the monosulfated disaccharides; (**c**) O/N ratio of the disulfated disaccharides. (*: *p* < 0.05). See Figure 1 for resolving the GAG structure codes.

A slightly higher relative abundance of the non-sulfated (D0A0) building block was observed in PCa than in BPH tissues (1.1-fold increase, *p* = 0,0094), with the parallel 16% decrease (*p* = 0.0022) of the D2A0/D0A6 unit (Figure 5a–d). A significantly lower level of the *N*-sulfated D0S0 building block was observed in the pTURP surgical group than in the RPE group (Figure 5e). Regarding the relations with the Gleason risk groups, we could conclude that all the sulfated building blocks except the D2A0/D0A6 showed a decreasing trend with increasing risk. The D0A0 disaccharide had a maximum abundance (Figure 5b), while the D2A0/D0A6 disaccharide pair had a minimum abundance in the IR PCa risk group (Figure 5d). This resulted in the average rate of sulfation (number of sulfate groups per disaccharide) showing a significantly lower value in the intermediate-risk group than in the BPH group (Figure A3a). Altered HS disaccharide content between the BPH and LR-PCa groups was observed in the case of D2A0/D0A6 isomers (9% decrease, *p* = 0.0411) and the D0S0 monosulfated disaccharide (11% increase, *p* = 0.0319). Other significant differences were not observed in connection with the risk groups (Figure 5g–l).

**Figure A4:**
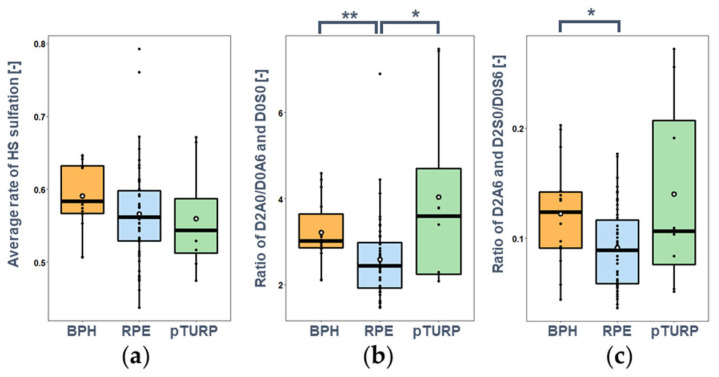
Sulfation characteristics of HS chains as a function of the type of surgery: (**a**) average rate of HS sulfation; (**b**) O/N ratio of the monosulfated disaccharides; (**c**) O/N ratio of the disulfated disaccharides. (*: *p* < 0.05, **: *p* < 0.01). See Figure 1 for resolving the GAG structure codes.

A change in the ratio of *N-*sulfation and *O-*sulfation was discovered as well. A similar trend to average sulfation was observed: Both for the monosulfated and the doubly sulfated disaccharides, the *O/N* ratio had a minimum level in the IR PCa group, while the high-risk and BPH groups had similar values, and the low-risk group was in-between (Figure A3b,c). A statistically significant difference was observed in the D2A0 + D0A6/D0S0 ratio between BPH and low-risk PCa (*p* = 0.0345) and between BPH and intermediate-risk PCa (*p* = 0.0280), but the differences in D2A6/D2S0 + D0S6 were not significant. As for the differences in operation type, a significantly higher (FC = 1.47, *p* = 0.043) *O/N* ratio was observed in the pTURP group for the monosulfated components in RPE; this trend was the same for the disulfated components but without statistical significance (Figure A4). 

### 3.4. Survival Analysis

**Figure 6:**
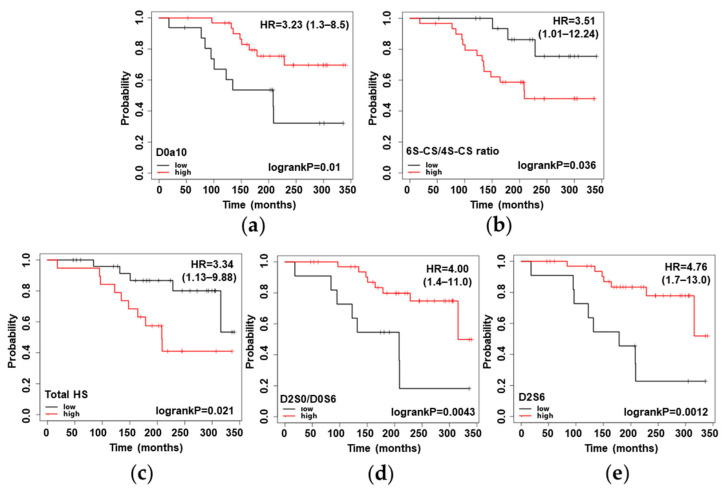
Kaplan–Meier plots of radical prostatectomy patients, based on the level of a given GAG motif: (**a**) D0a10 relative abundance; (**b**) 6S-CS/4S-CS ratio; (**c**) total HS abundance; (**d**) D2S0/D0S6 relative abundance; (**e**) D2S6 relative abundance. Low- and high-value groups were not further corrected for the Gleason score and pathological stage data. See Figure 1 for resolving the GAG structure codes.

Next, we investigated the survival rate of the RPE (*n* = 48 for CS and *n* = 46 for HS study) patients based on the abundance and sulfation pattern of the GAGs present in PCa tissue. CSS analysis was performed for each covariate (univariate Cox regression), and Kaplan–Meier plots were constructed. GAG motifs were dichotomized with an automatic cut-off into low and high levels. Significant differences in CSS probability could be observed for several CS and HS motif levels (Figure 6). 

In terms of CS, the relative abundance of the disulfated D0a10 disaccharide (HR = 3.23) and the ratio of the monosulfated disaccharides (6S-CS/4S-CS ratio, HR = 3.51) showed significant (log-rank *p* < 0.05) differences in CSS probability (Figure 6a,b). In terms of HS, groups with significantly different CSS probability could be formed based on the total HS content and the relative abundances of D2S0/D0S6 and D2S6 disaccharide building blocks, with hazard rates of 3.34, 4.00, and 4.76, respectively (Figure 6c–e). 

### 3.5. Independence of GAG Motifs from Currently Used Clinical Parameters

To assess whether the GAG motifs shown in Figure 6 could be independent predictors of survival, a multivariate Cox regression was fitted. The covariates were compared in a pairwise manner: each GAG motif and the three clinical parameters (T-stage, PSA level, and ISUP grade). The HS total intensity and the two CS motifs (relative abundance of D0a10 and 6S-CS/4S-CS ratio) did not prove to be independent predictors of CSS. On the other hand, the relative abundance of the doubly and triply sulfated HS disaccharides (D2S0/D0S6 and D2S6) proved to be a significant predictor for all the three currently used clinical parameters included in this analysis (Table 1).

## 4. Discussion

In this study, we assessed the glycosaminoglycan patterns in tissue samples of BPH and different risk groups of PCa. We found altered patterns of CS in concordance with the current risk assessment methods. These altered patterns were characterized by decreasing trends of D0a0, D0a4, and D0a10 but significantly increasing amounts of D0a6. These motifs might serve as new prognostic markers after further validation. The total amount and sulfation characteristics of heparan sulfate (e.g., D2S0/D0S6 and D2S6) showed few concordances with risk groups; however, they were well-associated with patients’ CSS and may, therefore, serve as prognostic markers contributing to avoid both over- and undertreatment of clinically localized PCa. The independent markers detected in heparan sulfate characteristics may open the way to new risk assessment methodologies for improving therapeutic decisions in PCa patients.

The total amount of CS chains was 1.75–1.82 higher in cancerous samples than in the BPH samples, the difference being significant only in the BPH vs. RPE comparison. This alteration has already been linked to the upregulation of growth factors (e.g., TGF-β) in androgen-dependent processes [22]. In the case of other cancer types, it has been shown that the changes in the stroma attributed to tumor progression are accompanied by the accumulation of CS chains. Its main cause is the overexpression of the *CHSY-1* gene coding chondroitin synthase-1 enzyme [23,24]. The results presented in this study corroborate previous findings that the increase in the quantity of CS chains might be an early stage marker of PCa progression, and there is a correlation between CS total abundance and the aggressivity of prostate tumors [25,26]. The increased CS content is also well in agreement with previous IHC studies, where Versican (CSPG) showed elevated levels in the stroma of PCa samples compared with those with BPH [27].

In terms of sulfation, we observed similar changes when comparing BPH with the cancerous tissue and within the tumor groups with different levels of severity. The non-sulfated (D0a0) disaccharide showed a smaller relative abundance on average in cancer and a small but steady decrease with the increasing Gleason scores. The same trend was observed for the D0a4 disaccharide; however, in this case, all the cancerous groups showed differences that were statistically significant as well. The D0a6 disaccharide, on the other hand, showed an increasing trend and significant differences with cancer progression. As a result of these changes, we observed an increased rate of average sulfation with cancer progression: 7.4% and 18.5% increase in IR and HR PCa, respectively, compared with LR PCa. The 6S/4S ratio showed the largest differences with the smallest *p*-values; almost all the groups showed significant differences between each other. This change in the dominant sulfation positions is of utmost importance; it has already been shown in other cancer types as well [28,29]. One possible underlying reason is the change in the activity of the chondroitin-4-sulfotransferase (C-4-ST) enzyme; however, the implementation of the data was not straightforward. The affected disaccharide building blocks (D0a4 and D0a10) both showed a decreasing tendency with the increasing Gleason risk groups. However, the change was smaller in the case of D0a10, and also an opposite trend was observed when comparing the BPH group to the LR PCa group. Thus, the underexpression of the *CHST11* gene (coding C-4-ST) is implicated but not proven by the results, and other factors altering the sulfatase activity may play a role during cancer progression. Another observation of our study was the large increase in the relative abundance of D0a6 implicating the increased activity of chondroitin-6-sulfotransferase (C-6-ST) enzyme or alterations in the expression of the genes *CHST3* and *CHST7* coding C-6-ST [30]. This hypothesis should be further tested in the future. Nevertheless, the presented results corroborate previous findings that a parallel increase in the total CS content and the ratio of 6-*O*-sulfation support cellular rearrangement and tumor progression [29], while the increased binding capabilities of CS chains (introduced by 6-*O*-sulfation) to most of the growth factors promote intercellular signaling [31]. Interaction with macrophages is one of the most important functions of CS changes for maintaining protumor–antitumor balance. The chondroitin-6-sulfate building blocks and distinct C-6-S chains suppress the release of inflammatory molecules from macrophages, thus inhibiting most of the inflammatory mediators and NF-κB activation. As a result, an increase in C-6-S helps to maintain the M2 polarization of the cells, increasing the chances of the survival of tumorous cells [32,33,34].

It is also interesting to look at the roles of the distinct proteoglycans bearing CS chains (CSPGs) [15] since the detected changes in CS quantity and sulfation inherently affect the functions of PGs. Versican, a major CSPG, plays an important role in cancer progression via regulating cell adhesion, migration, proliferation, differentiation, and angiogenesis through both its core protein and the structure of the attached CS chains [35,36]. Another important role of Versican is regulating the interactions between chemokines and selectins (e.g., stromal cell-derived factor-1β), thus having a key role in inflammation preceding BPH and PCa [37,38]. A study on PCa cell lines also showed that a versican-rich matrix might enable increased tumor cell motility and facilitate local invasion, and thus metastasis [39]. Decorin has mostly been known for its tumor-suppressor functions through TGF-β and EGFR-mediated signaling [40,41] and was previously found to be downregulated in PCa. Thus, we assume that the increase in CS levels is not directly associated with altered decorin expression. However, several mechanisms exist to support an inhibitory role for decorin in PCa development; thus, further immunohistochemistry and targeted proteomics studies are necessary in the future to validate our findings.

Comparing the heparan sulfate levels of RPE samples with those of BPH, on average, a 1.4-fold increase was observed, but the pTURP sample group gave almost identical median and mean values to the BPH group. When looking at the PCa samples according to the risk stratifications, an increase in the mean HS total intensity was observed in cancerous tissues compared with BPH. No statistically significant differences were observed in the PCa risk group comparisons regarding the total abundance of HS chains; however, a decreasing trend was observed.

Regarding sulfation motifs, significantly lower levels of the *N*-sulfated D0S0 building block were observed in the more progressed pTURP group than in the RPE group. Regarding the correlations with the Gleason risk assessment groups, we could conclude that all the sulfated building blocks except D2A0/D0A6 showed a decreasing trend with higher risk groups. A trend showing a minimum in the intermediate-risk group was observed for the average rate of sulfation and the *O/N* ratio in the case of both the monosulfated and the doubly sulfated disaccharides. Furthermore, a significantly higher *O/N* ratio was observed in the pTURP operation group for the monosulfated components; this trend was the same for the disulfated components but without statistical significance. It is important to note that the average rate of the sulfation of HS was lower and that of CS was higher with the increasing Gleason risk groups (and in PCa patients in general compared with BPH patients). Unfortunately, the actual functions of the different sulfation positions and domain structure of HS chains are still mostly unknown during malignant transformation. Moreover, there is an ongoing controversy in the literature regarding the overall sulfation changes in HS chains in various malignancies. For example, in the case of hepatocellular carcinoma, one study suggested undersulfation [42], while others have observed significantly decreased 6-*O-* and increased 3-*O*-sulfation, without a difference in the average rate of sulfation [43]. However, the lengths of the *N*-sulfated domains correlate with the concentration of the sulfate donor to NDST enzymes (3′-phosphoadenosine-5′-phosphosulfate, PAPS) [44]. The action of the NDST enzyme is described to be opposite to that of the EXT1/EXT2 heparan sulfate polymerase complex [45]. NDST1 can bind to EXT2, and the *N*-sulfation degree is affected by the level of EXT1 and EXT2 expression [46]. Those regulations, however, are only marginally reflected by our result in PCa; mainly the surgery groups show a large difference in *N/O* sulfation. The differences shown in this study may be attributed to the differential expression and structural alterations of the Syndecan HSPG family, as those have already been implicated as new prognostic markers in the literature [47,48,49,50,51].

As there were only minor alterations in HS structure as a function of the different risk groups of PCa, we hypothesized that GAG chains and their building blocks may be the independent predictors of CSS in current risk stratification methods; thus, they might be used for the additional classification of PCa patients. To validate this hypothesis, we performed pairwise multivariate comparisons of the individual GAG disaccharides and total GAG abundances with the currently used clinical parameters (ISUP grade, T-stage, and PSA level). The HS total intensity showed independence (*p* < 0.05) from the ISUP grade but not from the T-stage and PSA level. On the other hand, the relative abundance of the doubly and triply sulfated HS disaccharides (D2S0/D0S6 and D2S6) showed no correlation with any of the clinical parameters investigated. The relative abundance of D0a10 and the 6S-CS/4S-CS ratio proved to be slightly and highly dependent, respectively, on the investigated clinical parameters. The results are correlated with those of previous studies, such as the increased Versican (CSPG) expression shown in poorly differentiated tumors versus moderately differentiated carcinomas and Versican being an independent predictor of progression in patients with early stage cancer [27]. These findings are also in good correlation with those seen when linking GAG motifs to the Gleason risk groups. All the HS motifs and the relative abundance of D0a10 showed a small decreasing trend, and the 6S-CS/4S-CS ratio had a significant increase with the increasing risk groups. This is also reflected in the survival analysis, as in most cases, the lower level of a given GAG motif is associated with a lower probability of survival. The two exceptions were the 6S-CS/4S-CS ratio and the total amount of HS chains. The interpretation is straightforward for the CS isomer ratio due to the large significant increase in correlation with the current risk grouping. However, there is a bit of controversy in the case of the total HS amount, as we observed a decreasing trend with the increasing risk groups and its lower level predicts a higher probability of cancer-specific survival. In summary, we identified CS and HS motifs that could facilitate the survival assessment of the clinically localized PCa patients who underwent RPE. Out of these, the most promising ones are the relative abundance of doubly and triply sulfated HS disaccharides (D2S0/D0S6 and D2S6), which also proved to be the independent predictors of CSS compared with the currently used clinical parameters. Our results provide a solid basis for a further validation study on a larger independent cohort. 

This study has some limitations inherent to its retrospective nature, including potential selection bias (e.g., tissue availability from larger tumor samples). In addition, tumor-specific death in a clinically localized PCa cohort occurs in a relatively low rate of RPE-treated patients. This is also true for our cohort, which limited our statistical analysis, as we could only perform pairwise multivariate CSS analyses. The strength of our study is its large-scale disease spectrum (BPH and the low-, intermediate-, and high-risk localized as well as progressed (pTURP) stages of the disease). A further strength of this study is the availability of a long follow-up, which is necessary for the survival analysis of clinically localized PCa. 

## 5. Conclusions

In this study, we presented, for the first time, a clinically relevant correlation between GAG abundance and sulfation changes, and PCa progression. In addition, we found that altered heparan sulfate patterns were associated with poor patient survival in clinically localized PCa. The relationships that we observed in chondroitin sulfate analyses suggest these motifs as new prognostic markers and potential therapeutic targets for the targeted biological therapy of PCa. The independent markers detected in heparan sulfate characteristics may open the way to new risk assessment methodologies for making therapeutic decisions in PCa patients more efficient. However, this requires the validation of our results in a large number of cases. In the case of successful validation, we plan to conduct studies to determine whether the detected markers can be measured through non-invasive sampling (e.g., urine) so that they can be easily implemented into clinical practice.

## Figures and Tables

**Table 1:** Multivariate analysis between clinical parameters (T-stage, ISUP grade, and PSA level) and GAG motifs (total HS, D2S0/D0S6, D2S6, D0a10 relative abundance, and 6S-CS/4S-CS ratio), FDR controlled *p*-values, and hazard ratios with 95% confidence intervals are presented. See Figure 1 for resolving the GAG structure codes.

	Total HS		D2S0/D0S6		D2S6		D0a10		6S-CS/4S-CS Ratio	
Clinical Parameter	*p*-Value	HR	*p*-Value	HR	*p*-Value	HR	*p*-Value	HR	*p*-Value	HR
T-stage	0.0573	0.36 (0.13–1.00)	0.0252	3.97 (1.32–11.92)	0.0152	4.63 (1.59–13.54)	0.0525	3.30 (1.23–8.85)	0.1807	0.40 (0.13–1.27)
PSA level	0.1595	0.45 (0.15–1.37)	0.032	3.43 (1.20–9.82)	0.0252	3.90 (1.34–11.29)	0.1807	2.31 (0.84–6.37)	0.2294	0.50 (0.16–1.56)
ISUP grade	0.0456	0.33 (0.12–0.93)	0.0151	5.17 (1.73–15.51)	0.0026	8.72 (2.70–28.16)	0.0525	3.45 (1.31–9.04)	0.2063	0.45 (0.15–1.41)

## Data Availability

The data presented in this study have been deposited in the GlycoPOST database [52] under the accession number GPST000295.

## Supplementary Materials

The following are available online at https://www.mdpi.com/article/10.3390/cancers14194867/s1, Table S1: Patient data of the full cohort on BPH and PCa. Table S2: Data analysis parameters for the evaluation of HPLC–MS results. Table S3: Statistical test performed for all the group comparisons presented in the Results section. Table S4: Summary of the measurement data (median and range values) according to different group classifications. CS and HS data are shown on separate sheets.
